# Microbial bioherbicides: Integrating bioprocessing, mechanisms of action and protein engineering for sustainable weed management

**DOI:** 10.1007/s11274-026-05366-2

**Published:** 2026-10-29

**Authors:** Daniela Dal Castel Krein, Angelo Antonio Maraschin Conterato, Ricardo Plucinski, Guilherme Luiz Dotto, Aline Dettmer

**Affiliations:** 1 Chemical Engineering Department, Postgraduate Program in Chemical Engineering (PPGEQ), Federal University of Santa Maria (UFSM), Santa Maria, RS Brazil; 2 Chemical Engineering Course, University of Passo Fundo (UPF), Technology Institute (ITEC), Passo Fundo, RS Brazil; 3 School of Agricultural Sciences, Innovation and Business (ESAN), Postgraduate Program in Food Science and Technology (PPGCTA), University of Passo Fundo (UPF), Passo Fundo, RS Brazil

**Keywords:** Bioprospecting, Biopesticides, Gene expression, Protein engineering

## Abstract

The widespread use of synthetic herbicides in agriculture has intensified concerns regarding environmental sustainability and human health, while the evolution of herbicide resistance has outpaced the discovery of new modes of action. Bioherbicides have consequently emerged as a viable alternative, comprising biologically derived compounds capable of suppressing weed growth. This review adopts an integrated perspective on microbially derived bioherbicides, following the active biomolecules from the producing strain and its fermentation conditions to their physiological targets in the plant and to the molecular strategies available for their optimization. Unlike previous reviews, which address these dimensions separately and focus mainly on secondary metabolites, particular emphasis is placed here on enzymes with herbicidal activity and on protein engineering approaches. Based on a structured literature search, 39 original studies were analyzed. Phytopathogenic fungi, mainly *Fusarium*, *Diaporthe*, *Phoma*, *Alternaria* and *Trichoderma*, predominate as producing organisms, followed by bacteria of the genera *Streptomyces*,* Pseudomonas* and *Bacillus*, and submerged fermentation using low-cost agro-industrial substrates is the prevailing production strategy. Enzymatic classes such as cutinases, lipases, cellulases, pectinases and proteases act primarily by degrading the cuticle and the cell wall, whereas secondary metabolites frequently target the photosynthetic apparatus, particularly photosystem II; most of these compounds, however, still lack a defined mode of action within established herbicide classification systems. Molecular approaches, including heterologous expression, molecular docking and metabolic engineering, remain comparatively underexplored despite their potential to enhance activity, stability and specificity. Advances in formulation stability, bioprocess scalability, mechanistic characterization and regulatory harmonization are identified as the key requirements to translate these biomolecules into commercially viable products for sustainable weed management.

**Graphical abstract:**

**Figure:**
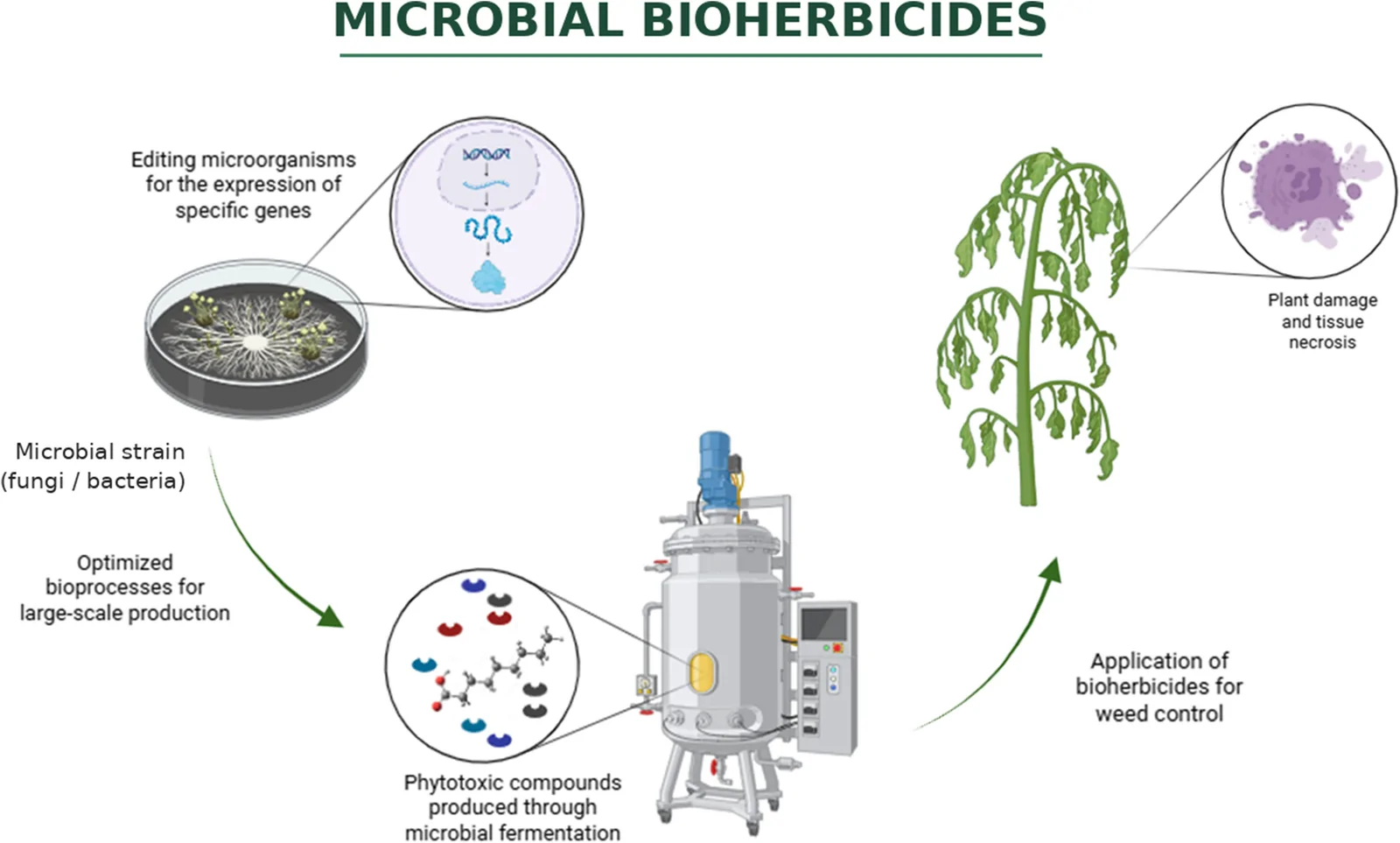

## Introduction

Weeds are among the main biology problem to crop production, causing yield losses primarily through competition with crops for essential resources such as light, water and nutrients. Fast-growing weeds can shade the crop canopy and reduce the photosynthetically active radiation available to crop plants, while their often extensive and aggressive root systems deplete soil water and mineral nutrients, particularly nitrogen, phosphorus and potassium, limiting their availability during critical stages of crop development. Competition for water is especially detrimental under drought conditions, when weed water uptake can intensify crop water stress and further reduce yield. In addition to resource competition, weeds can alter light quality (e.g., by lowering the red: far-red ratio) and release allelopathic compounds, triggering stress and shade-avoidance responses in crops even before resources become limiting, thereby suppressing their growth (Horvath et al. 2023).

To reduce invasive plant infestation, herbicide-based weed management has become one of the most widely adopted strategies in modern agriculture. The global herbicide market exceeds USD 45 billion, and resistance has already been reported for most known herbicide modes of action, highlighting the urgent need for alternative weed control strategies (Mordor Intelligence 2025; Heap, 2025). The cumulative number of confirmed herbicide-resistant weed cases has increased sharply over the last four decades, whereas the introduction of novel herbicidal chemistries has remained relatively stagnant. This divergence reflects a growing imbalance between the rapid evolution of resistance and the availability of innovative weed control tools. The widespread reliance on a limited number of herbicide modes of action has intensified selection pressure in agricultural systems, accelerating the emergence of resistant biotypes and compromising the long-term sustainability of chemical weed management.

This scenario has renewed interest in biologically based weed management strategies, particularly those relying on natural products and microbial metabolites. Unlike conventional herbicides, these compounds often exhibit complex chemical structures and may act through mechanisms distinct from those of currently available synthetic herbicides, potentially reducing cross-resistance and providing valuable tools for managing herbicide-resistant weed populations. Bioherbicides based on such compounds offer several further advantages. Being derived from natural sources, they are generally biodegradable and exhibit short environmental persistence, resulting in reduced residues in soil, water and harvested products. Many of them display high target specificity, minimizing damage to crops and non-target organisms, such as beneficial insects, soil microbiota and wildlife, and typically present lower toxicity to humans and animals, which improves applicator safety and often allows shorter re-entry and pre-harvest intervals. They are also compatible with integrated weed management programs and, in many cases, with organic production systems, in which synthetic herbicides are restricted or prohibited. From a regulatory and economic perspective, biopesticides generally undergo faster and less costly registration processes than conventional chemical products, and microbial bioherbicides can be produced from renewable raw materials, including agro-industrial residues, in line with circular bioeconomy principles. Moreover, they meet the growing consumer and market demand for food produced with lower chemical inputs (Dayan and Duke 2014; Cordeau et al. 2016; Hasan et al. 2022). For these reasons, bioherbicides represent a sustainable approach to weed control while also expanding the repertoire of bioactive molecules and molecular targets available for herbicide development.

Bioherbicides defined as microbial or plant origin whose activity relies on enzymes, secondary metabolites, and other bioactive molecules produced by the source organism have emerged as a promising alternative (Hasan et al. 2022). Despite this potential, the adoption of biological weed control remains slower than that of other bioinput categories, such as bioinsecticides, bionematicides, and biofungicides, which accounted for 35%, 30%, and 22%, respectively, of the bioinput market value in Brazil in 2025 (CropLife, 2026). The adoption of bioherbicides faces substantial challenges, primarily related to the complexity of their production bioprocess: the development of this technology depends on the selection of an appropriate species, the optimization of fermentation parameters, and the refinement of downstream processing steps, all of which are essential to ensure a viable and efficient formulation. Compounding this challenge is the limited understanding of the mechanisms of action and specificity profiles of microbially derived bioherbicides, which constrains their development and may reduce their effectiveness under field conditions (Zhang et al. 2025).

Within this landscape, microbial bioherbicides emerge as a particularly promising yet underexplored strategy. Compared with their plant-derived counterparts, they offer relevant comparative advantages, including greater scalability through industrial fermentation, more consistent and controllable production relative to plant compound extraction, and the possibility of genetically engineering the producing strain to optimize yield and specificity (Ocán-Torres et al. 2024; Camargo et al. 2023). Furthermore, there is limited knowledge regarding the persistence of allelopathic molecules in the soil, the primary compounds responsible for the bioherbicidal activity of plant extracts, and their possible carryover phytotoxicity to subsequent crops remains to be clarified (Scavo et al. 2019; Fadiji et al. 2025). In view of these aspects, microbially derived bioherbicides are the focus of this review.

Several reviews on bioherbicides have been published in recent years, each addressing a specific dimension of the field. Some provide global overviews of available products and commercialization bottlenecks (Triolet et al. 2020; Roberts et al. 2022; Hasan et al. 2021; Duke 2024); others focus on the chemical diversity of fungal phytotoxins and secondary metabolites with herbicidal activity (Cimmino et al. 2015); and more recent contributions have systematized the physiological modes of action of bioherbicidal compounds (Zhang et al. 2025) or the use of cell-free metabolites in weed control (Ocán-Torres et al. 2024). Despite their individual contributions, these reviews address the production bioprocess, the mode of action and the molecular optimization of the active biomolecules as largely separate topics, and most of them concentrate on secondary metabolites, with limited attention to the enzymatic fraction responsible for the degradation of plant structural barriers. The present review differs from these contributions in three respects. First, it adopts an integrated perspective that follows the active biomolecules along the entire development chain, from the selection of the producing strain and the fermentation conditions that govern their synthesis to their physiological targets in the plant. Second, it places particular emphasis on enzymes with herbicidal activity, such as cutinases, pectinases, cellulases and proteases, whose role in cuticle and cell wall degradation has been comparatively neglected in previous reviews. Third, it incorporates protein engineering and heterologous expression as explicit tools for improving the activity, stability and specificity of these biomolecules, a dimension that the keyword co-occurrence analysis presented in Section “Bibliometric analysis” identifies as peripheral in the current literature.

Accordingly, this review aims to provide an integrated overview of microbial bioherbicides, connecting bioprocessing, mechanisms of action and protein engineering. Specifically, it seeks to (i) describe the main microbial sources of bioherbicidal molecules and the fermentation strategies and culture conditions used to obtain them; (ii) discuss the mechanisms of action of the enzymes and metabolites responsible for phytotoxicity, with emphasis on their effects on plant metabolic and structural processes; (iii) examine how protein engineering and modern molecular tools can be applied to enhance the activity, stability and specificity of these biomolecules; and (iv) identify the main challenges that currently limit the development and commercialization of microbial bioherbicides, outlining perspectives for future research. By bringing these aspects together, this review intends to contribute to a more comprehensive understanding of how microbially derived biomolecules can be developed into effective tools for sustainable weed management.

## Bibliometric analysis

The search was performed in March 2025 in the Scopus and PubMed databases and in the Wiley Online Library, with the aim of identifying original studies reporting the production, identification or optimization of microbially derived biomolecules with herbicidal activity. The following search string was applied to title, abstract and keywords: (“bioherbicide” OR “biological herbicide”) AND (protein* OR peptide* OR enzym* OR toxin*) AND (microb* OR fung* OR bacter*) AND (“discovery” OR “screening” OR “protein engineering” OR “recombinant protein”). The string was structured in four blocks combined by the AND operator, each delimiting one dimension of the scope of this review: the subject area, including the two designations most commonly used for this class of products; the biomolecules of interest, restricting the results to studies on the compounds responsible for the herbicidal activity rather than on the application of whole organisms; the microbial origin of these molecules, excluding plant-derived allelochemicals; and the development stages addressed here, from prospection and screening to molecular approaches for improving the biomolecules. Truncation (*) was used to retrieve lexical variants of each term.

In Scopus, the string was applied using the TITLE-ABS-KEY field code; in PubMed, the Title/Abstract field tag was used; and in the Wiley Online Library, the search was restricted to title, abstract and keywords. Scopus and PubMed were selected because they provide complementary coverage of the fields relevant to this review: Scopus offers broad multidisciplinary indexing of agricultural sciences, biotechnology and bioprocess engineering, while PubMed concentrates the biomedical and microbiological literature, including studies on microbial metabolites, enzymes and protein engineering that are not always retrieved by agronomy-oriented sources. The Wiley Online Library was additionally consulted as a publisher platform in order to capture full-text records from relevant journals in plant science and biotechnology, complementing the indexed searches. The initial search retrieved 777 records in Scopus, 112 in the Wiley Online Library and 324 in PubMed, totaling 1,213 records.

Records were restricted to documents published between 2005 and 2025, reducing the sample to 668, 92 and 287 records in Scopus, Wiley and PubMed, respectively. A second filter was then applied for document type and language, retaining only original research articles written in English and excluding reviews, book chapters, conference papers, editorials and patents. After this stage, 378 records remained from Scopus, 40 from Wiley and 249 from PubMed, totaling 667 records.

**Fig. 1:**
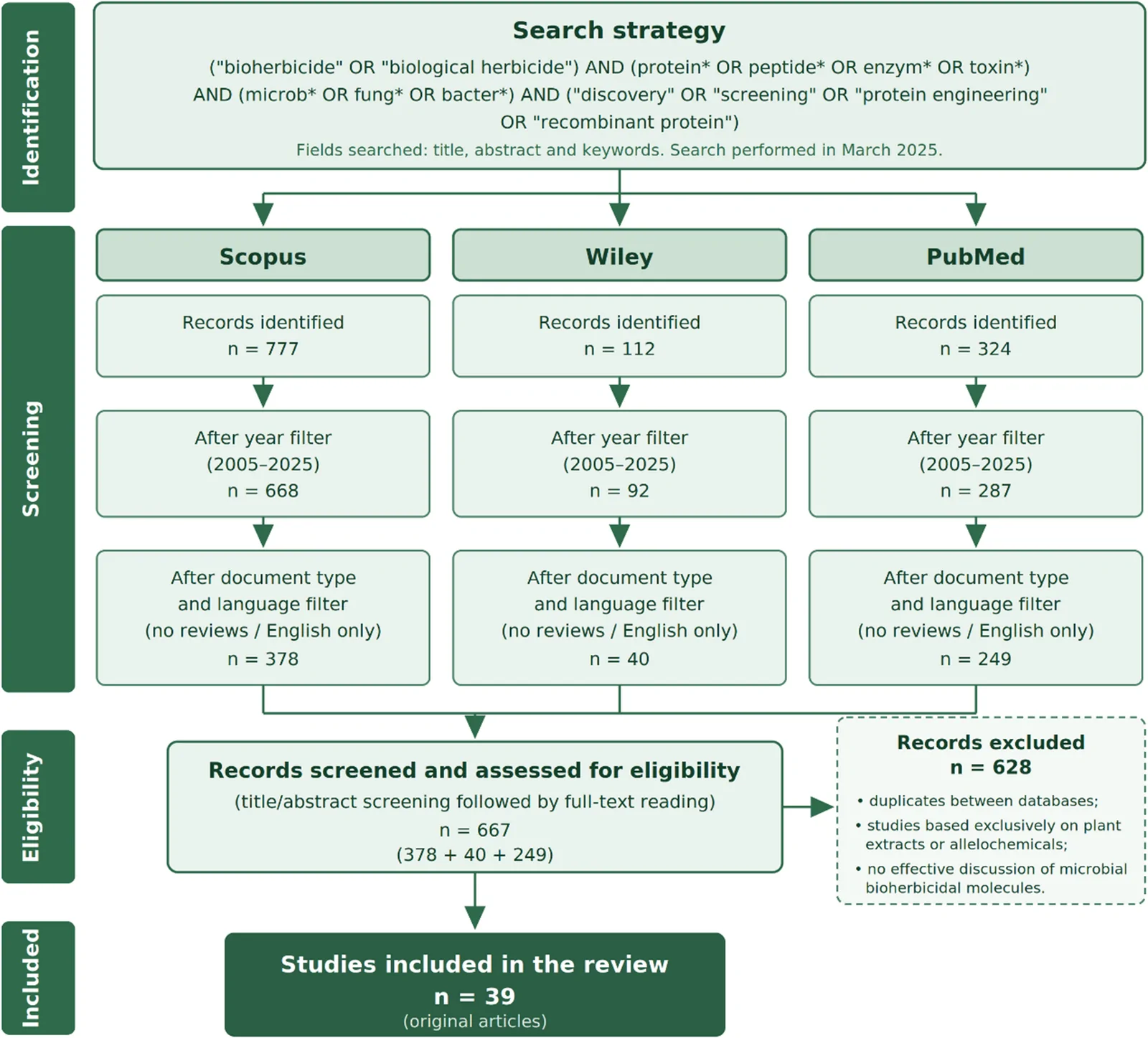
PRISMA flow diagram of the identification, screening and selection of the studies included in this review

These records were subsequently screened by title and abstract, and all studies based exclusively on plant extracts or plant-derived allelochemicals were discarded, since the scope of this review is restricted to microbially derived bioherbicides. The remaining records were read and analyzed in full text and were retained only when they contained effective discussion of the topics defined by the search terms, namely the production of proteins, peptides, enzymes or toxins of microbial origin with herbicidal activity, or the discovery, screening and engineering of such biomolecules. Records in which these terms appeared only incidentally, without substantive discussion of microbial bioherbicidal molecules, were also excluded. Duplicate records retrieved from more than one source were counted only once. A total of 628 records were excluded across these stages, and 39 original articles met all criteria and were included in the review (Fig. 1).

For each included study, the following information was extracted: microbial source, culture medium, fermentation type and operational conditions, bioactive molecule or enzyme identified, target weed species, concentration applied, and reported phytotoxic effects. The extracted data were organized into thematic categories corresponding to the sections of this review: microbial sources and fermentation strategies, mechanisms of action of the bioactive molecules, and protein engineering approaches for bioherbicide optimization. A qualitative synthesis was then carried out, and the fermentation parameters and phytotoxic responses reported in the selected studies were compiled in Table 1.

**Table 1:** Parameters and culture media used for the fermentation of microorganisms with bioherbicidal potential

Microorganism	Culture medium	Fermentation conditions	Results	Author(s)
*Nigrospora sphaerica*	Potato dextrose agar	SSF; Temperature at 25 °C; Incubation time of 14 days;	At 2 mg/mL, the crude culture filtrate extract caused complete failure of germination of *Cenchrus ciliaris* seeds (100% inhibition) in the coleoptile and radicle elongation bioassay, whereas the untreated control germinated at > 95%.	Salvatore et al. (2024)
*Bacillus altitudinis strain* D30202	Culture medium ATCC213 modified (0.2 g CaCl_2_·2H_2_O, 10 g MgSO4·7H_2_O, 2.5 g peptone, 5 g KCl, 30 g NaCl and 10 g baking powder	SbF	Genome mining identified enriched pathways for 4-hydroxy-3-methoxycinnamic acid and two indole derivatives with reported bioherbicidal activity against *Avena fatua* L.; phytotoxicity percentages were not reported in this study.	Ma et al. (2023)
*Streptomyces* sp. strain KR0005	1% soybean, 1% glucose, 2% starch, 0.3% CaCO_3_, 0.02% FeSO_4_ and 0.1% antifoaming agent	SbF; Temperature at 25 °C; Incubation time of 7 days;	The broth culture caused > 96% growth reduction in *Digitaria ciliaris*, with a GR50 of 1.37 g/L of culture filtrate; at 10 g/L, fresh weight was reduced by more than 85%. Cycloheximide acted as a dose-dependent inhibitor of photosynthesis and cell membrane integrity, reducing chlorophyll content by 36% at 2 g/L.	Umurzokov et al. (2023)
*Streptomyces* sp. NEAU-HV44	1% malt extract, 0.4% glucose, 0.4% yeast extract, 2% starch and 1 mL/L trace elements solution	SbF; Temperature at 28 °C; Incubation time of 7 days;	At 2 mg/mL (post-emergence), the supernatant extract significantly reduced plant height and caused more than 50% biomass (fresh weight) reduction in *A. retroflexus* L., *Setaria viridis*, *Portulaca oleracea* L. and *Chenopodium album*; in pre-emergence, 0.2 mg/mL inhibited shoot and root growth of *A. retroflexus* L. by 92.31% and 93.71%, respectively.	Ling et al. (2023)
	For fungi: 10 g/L glucose, 10 g/L yeast extract, 2 g/L peptone, 0.5 g/L (NH_4_)_2SO4_, 1 g/L FeSO4·7H2O, 1 g/L MnSO4· H2O and 0.5 g/L MgSO4 For bacteria: 1 g/L beef extract, 5 g/L peptone, 2 g/L yeast extract and 5 g/L NaCl	SbF; Temperature at 25 °C; Incubation time of 7 days;	With 1 mL of crude broth per plant, phytotoxicity in *C. sativus* reached 90% for *N. sphaerica* (A14.2 and B22.2) and 80% for *B. velezensis* (B14) and *A. flavus* (γ) at 21 DAA. In weeds, B14 reduced plant height of *E. crus-galli* by 19% and shoot and root fresh and dry mass by 52–67%.	Schein et al. (2022)
*Diaporthe schini*	20 g/L sucrose and 15% corn maceration liquor	SbF; Temperature at 28 °C; Incubation time of 7 days;	Applied post-emergence at 200 L/ha, the broth obtained by submerged fermentation provided higher phytotoxicity than solid-state fermentation at 14 DAA: 38% vs. 12% in *B. pilosa*, 32% vs. 14% in *A. viridis* and 28% vs. 12% in *E. crus-galli*, with height reductions of up to 71% in *L. multiflorum*.	Brun et al. (2022a)
*Diaporthe schini*	SbF medium culture: 10 g/L glucose, 2 g/L yeast extract, 7.5 g/L peptone, 1 g/L (NH_4_)_2_SO_4_, 1 g/L FeSO_4_·7H_2_O, 1 g/L MnSO_4_· H_2_O and 0.5 g/L MgSO_4_;	SbF; Temperature at 28 °C; Incubation time of 7 days;	Powder obtained by spray drying with lactose, applied at 10% (w/v) and 200 L/ha, produced herbicidal activities of 38%, 45%, 21% and 18% and height reductions of 69.0%, 74.3%, 20.4% and 24.8% in *B. pilosa*, *A. viridis*, *E. crus-galli* and *L. multiflorum*, respectively.	Brun et al. (2022b)
	SSF medium culture: rice, rice bran, rice husk, sugarcane bagasse, and malt bagasse with different humidity percentile	SSF; Temperature at 28 °C; Incubation time of 7 days;		
*Trichoderma koningiopsis* and *Rhizopus stolonifer*	10% orange peels and 0.3% shrimp shell	SbF; Temperature at 28 °C; Incubation time of 3 days;	With 5 mL of undiluted extract applied per plant, leaf damage in *C. sativus* reached 66.67% (*T. koningiopsis*) and 77.08% (*R. stolonifer*) at 7 days, rising to 75.02% and 79.16% at 15 days; extracts showed high peroxidase activity (101.88 and 95.32 U/mL).	Cavalcante et al. (2021)
*Alternaria cassiae*	Soybean flour and corn flour	Unavailable	Following inoculation with 10⁵ spores/mL, pectinase activity increased from 16 h after application, reaching 1.5-fold above control in light-grown and 2-fold in dark-grown sicklepod seedlings, suggesting that increased pectinase activity during pathogenesis acts as an infectivity mechanism by degrading pectic polymers important for cell wall integrity; phytotoxicity percentages were not reported.	Hoagland and Boyette (2021)
*Phoma* sp.	200 g/L potato, 20 g/L glucose, 2 g/L yeast extract, 10 g/L peptone, 2 g/L (NH_4_)_2_SO_4_, 1 g/L FeSO_4_·7H_2_O, 1 g/L MnSO_4_· H_2_O and 0.5 g/L MgSO_4_	SbF; Temperature at 28 °C; Incubation time of 7 days;	With 2 mL applied to detached *C. sativus* leaves, the crude cell-free broth (6.62 g/L of exopolysaccharides) caused 100% yellowing, while the microfiltration retentate (9.18 g/L) caused chlorosis (30.4%), necrosis (48.2%) and yellowing (21.4%), leading to leaf destruction without recovery.	Luft et al. (2021)
*Fusarium fujikuroi*	10 g/L sucrose, 50 g/L corn maceration liquor, 0.5 g/L (NH_4_)_2_SO_4_, 1 g/L FeSO_4_·7H_2_O, 1 g/L MnSO_4_· H_2_O and 0.5 g/L MgSO_4_	SbF; Temperature at 31 °C; Incubation time of 7 days;	With 2 mL applied to detached *C. sativus* leaves, the concentrated broth (5.94 g/L of exopolysaccharides) caused necrosis (5.85%), chlorosis (16.66%) and slight depigmentation (77.79%); lyophilization (10.64 g/L) resulted in 100% total leaf depigmentation.	Todero et al. (2020)
*Streptomyces anulatus*	10 g soybean, 10 g glucose, 20 g soluble starch and 3 g CaCO_3_	SbF; Temperature at 25 °C; Incubation time of 7 days;	In pre-emergence, the fermentation broth reduced seed germination by 90.4% in *Digitaria sanguinalis*, 81.3% in *Sorghum bicolor* and 85.2% in *Solanum nigrum*; in post-emergence, 100% control of grass and broadleaf weeds was achieved at the 1x concentration (5 mL sprayed on the foliage).	Bo et al. (2019)
*Fusarium acuminatum*,* F. verticillioides*,* F. equiseti* and *F. oxysporum*	Rice bran, soybean meal (2.5%) and corn maceration liquor (2.5%)	SSF; Temperature at 28 °C; Incubation time of 5 days;	Maximum cutinase yield of 16.22 U/g of substrate; the formulation containing 250 U of cutinase plus bioherbicide showed the highest herbicidal efficiency against *Conyza* sp. and *Ipomoea* sp., higher than the bioherbicide alone. Phytotoxicity percentages were not reported.	De Oliveira et al. (2019)
*Trichoderma* sp. strain T23, *Trichoderma* sp. strain T16) and *Paecilomyces* sp. PS1 strain	Malt extract agar	SSF; Temperature at 25 °C; Incubation time of 7 days;	*Trichoderma* sp. strain *T23* was responsible for a control of 82% under the species *F. graminearum*. The authors associate control due to the presence of a secondary metabolite identified as 6-pentyl-alpha-pyrone.	El-Hasan et al. (2018)
*Pseudomonas aeruginosa* C1501	For bacteria: 4 g NaHPO_4_, 2 g KHPO_4_, 0.8 g (NH4)_2_SO_4_, 0.1% yeast extract, 0.8 g MgSO_4_, 3% glycerol; For fungi: 2% glucose, 2% malt extract and 0.1% peptone.	SbF; Incubation time of 3 days;	Rhamnolipid at 0.003% v/v combined with the phytotoxic metabolite of *L. pseudotheobromae* C1136 enhanced biodegradation of *A. hybridus* L. and *E. crus-galli* and induced necrosis; the highest enzyme activities were 35 U/mL of xylanase and 21 U/mL of CMCase, against 7 and 8 U/mL in the controls.	Adetunji et al. (2017)

The systematic search was restricted to original research articles, which constitute the primary evidence base analyzed in this review. Review papers, books and database records were not retrieved through this search and are therefore not represented in the PRISMA flow diagram; they were consulted separately and are cited throughout the text to support the interpretation and discussion of the findings obtained from the original studies, providing conceptual background on topics such as fermentation regulation, enzyme function and herbicide classification.

**Fig. 2:**
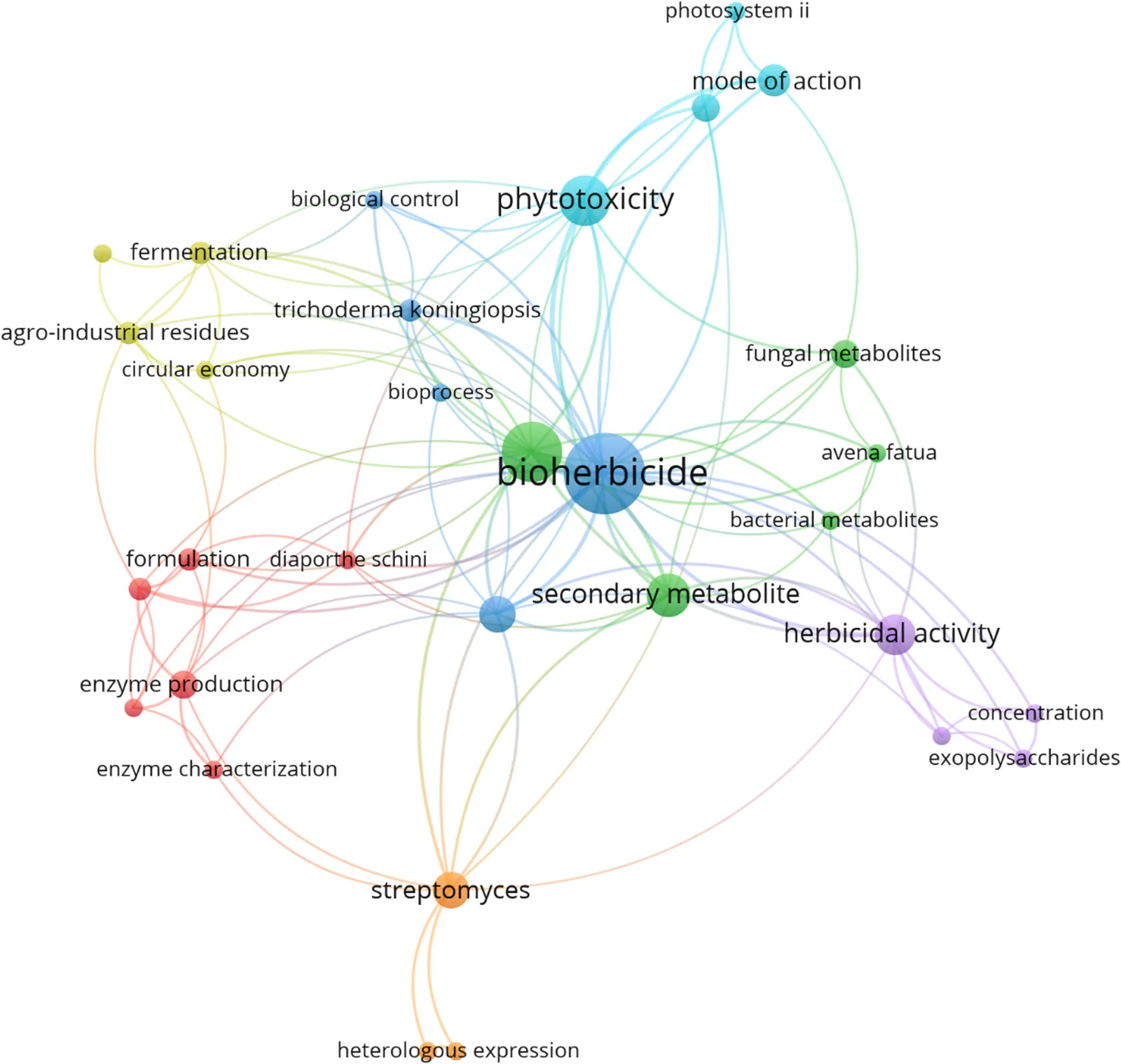
Co-occurrence network of the keywords of the 39 original articles included in this review, generated with VOSviewer

A co-occurrence analysis of the keywords of the 39 selected articles was performed using VOSviewer (version 1.6.20). The analysis was based on all keywords, with a minimum number of occurrences of two per term, and the resulting network was visualized as a co-occurrence map in which the size of each node is proportional to the frequency of the term, the thickness of the links represents the strength of co-occurrence between terms, and colors indicate the clusters identified by the software’s association-strength clustering algorithm (Fig. 2).

The co-occurrence map (Fig. 2) shows a network organized around the term bioherbicide, which presented the highest frequency and the largest number of links, confirming it as the central concept connecting all thematic groups. Around this core, the clustering algorithm identified groups that correspond closely to the stages of bioherbicide development addressed in this review, indicating that the selected literature covers the entire chain from microbial production to mode of action.

One cluster groups terms related to bioprocessing, such as fermentation, agro-industrial residues and circular economy, reflecting the predominance of studies that seek to reduce production costs through the use of low-cost substrates. A second cluster associates enzyme production, enzyme characterization and formulation, revealing a line of research focused on the enzymatic fraction of the fermented broth and on its stabilization into applicable products. A third cluster connects phytotoxicity, mode of action and photosystem II, corresponding to studies that investigate the physiological targets of the active molecules, with a marked concentration on the photosynthetic apparatus. A fourth cluster brings together secondary metabolite, fungal metabolites and bacterial metabolites, together with target species such as Avena fatua, representing the prospection of bioactive compounds and the evaluation of their herbicidal spectrum. Finally, a smaller cluster links Streptomyces and heterologous expression, which corresponds to the molecular and protein engineering approaches.

Two aspects of the map deserve attention. First, the strong connection between the bioprocessing and the metabolite prospection clusters indicates that most studies address production and phytotoxic activity jointly, whereas the terms related to genetic and protein engineering appear peripherally and with fewer links, confirming that this is still an underexplored dimension of microbial bioherbicide research. Second, terms associated with field application, formulation stability and environmental fate are scarce or absent from the network, which is consistent with the technological bottlenecks discussed in Section “Challenges and Future Perspectives” and reinforces the gap between laboratory-scale phytotoxicity studies and commercially viable products.

The following sections are organized accordingly. The first describes the microbial sources of bioherbicidal molecules and the fermentation strategies and culture conditions that govern their production. The second examines the mechanisms of action of the enzymes and secondary metabolites responsible for phytotoxicity, from the degradation of the structural barriers of the plant to the inhibition of the photosynthetic apparatus. The third addresses the molecular and protein engineering approaches applied to enhance the activity, stability and specificity of these biomolecules. The last discusses the technological and regulatory bottlenecks that still limit the development of commercial products, outlining perspectives for future research.

## Microbial sources of bioherbicide molecules

According to Ocán-Torres et al. (2024), the development of bioherbicides via microbial fermentation can be categorized into four distinct stages. The authors systematize the process in: (I) isolation and initial screening of the microorganism; (II) characterization of metabolites and optimization of the formulation; (III) greenhouse toxicity assessment; and (IV) scaling and purification of the bioinput.

**Fig. 3:**
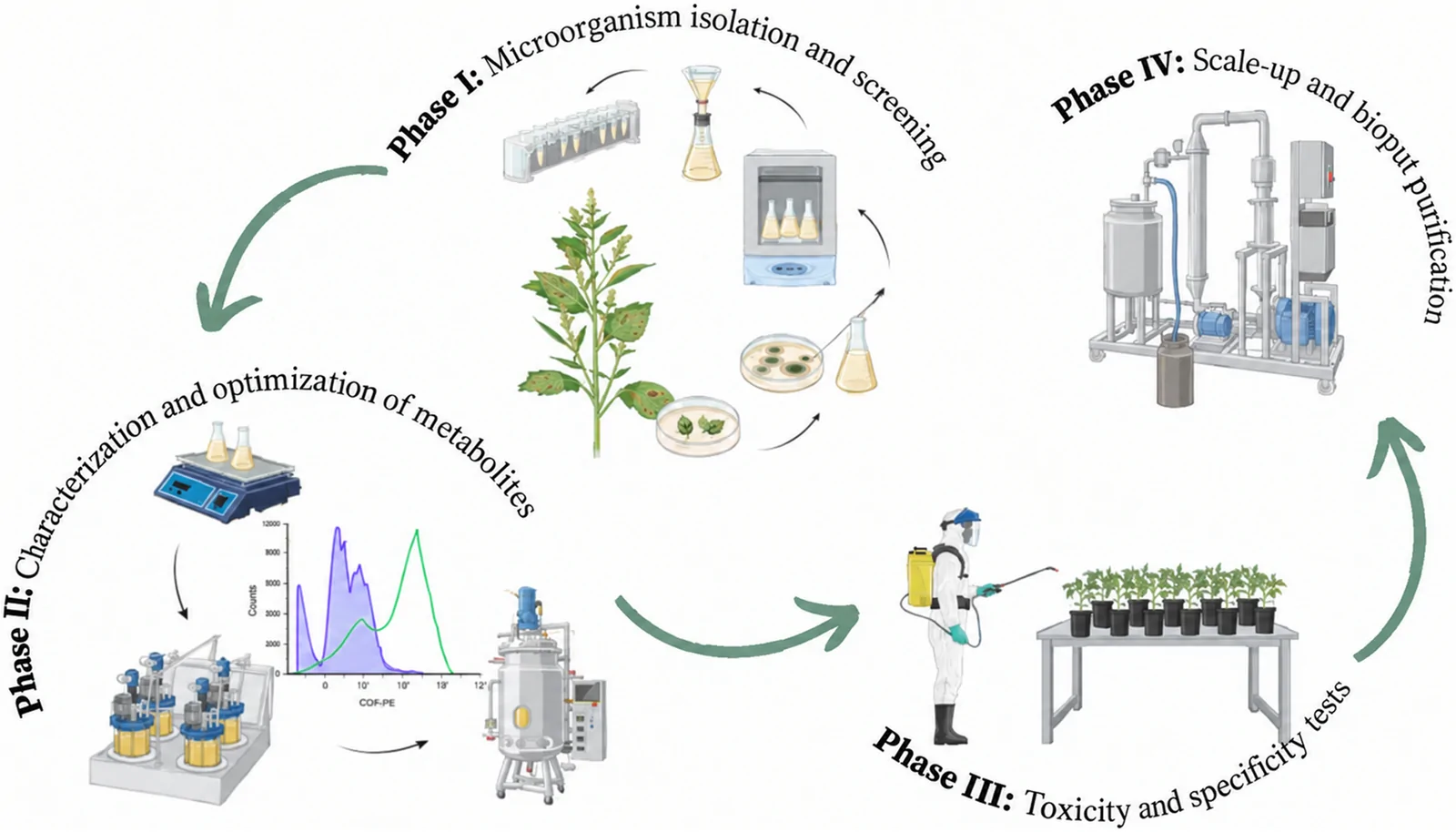
Stages of development of bioherbicides based on the fermentation of microorganisms

As illustrated in Fig. 3, this flow highlights the application of purified biomolecules, to the detriment of the use of fermented broths containing free cells. The preference for the use of metabolites rather than propagules — especially pathogenic genera such as *Fusarium* spp. and *Aspergillus* spp.— should be prioritized. This approach not only mitigates environmental and product drift risks in the field, but also optimizes weed control and reduces interference with non-target crops (Ocán-Torres et al. 2024).

The isolation of microorganisms from leaves or the rhizosphere with necrotic spots is a fundamental step in the screening of phytotoxic agents capable of damaging plant structures (Islam et al. 2024). By infecting the host, these phytopathogens interfere with the plant’s physiology, either by secreting enzymes that degrade the cell wall or by deregulating cell metabolism. The observation of symptoms such as chlorosis and necrosis indicates the impairment of chlorophyll synthesis, which impairs the capture of light energy and, consequently, inhibits photosynthetic activity (Cheaib and Killiny 2025). Therefore, the screening of microorganisms that induce such symptoms contributes to the identification of biomolecules with biotechnological potential for large-scale application, such as bioherbicides.

Studies that address the prospection of microorganisms for application as bioherbicides are mostly based on studies with phytopathogenic fungi, being the genera *Fusarium* spp., *Diaporthe schini*,* Phoma* spp., *Alternaria* spp. and *Trichoderma* spp., the most cited in the literature (Bordin et al. 2018; Brun et al. 2022a; Cavalcante et al. 2021; Liu et al. 2024; Luft et al. 2021; Schmaltz et al. 2025). Bacteria of the genus *Pseudomonas* (Adetunji et al. 2017; Mupondwa et al. 2015), *Streptomyces* (Ling et al. 2023; Umurzokov et al. 2023; Wang et al. 2022) and *Bacillus* (Ma et al. 2023) appear with some frequency, but correspond to a lower percentage of research when compared to fungal species. Fungi-based research is found in greater numbers, since they may present greater resistance to bacteria in terms of specificity to the host plant, avoiding damage to non-target plants and extracellular enzymes which are capable of depolymerizing the structural components of plant cells (Kubicek et al. 2014).

The production of bioherbicides depends on the optimization of the bioprocess for its obtainment, and the choice of the fermentation technique is a determining factor for economic viability. The most well-known methods are Submerged Fermentation (SbF), characterized by the growth of microorganisms in liquid media, and Solid-State fermentation (SSF), in which cultivation takes place in a solid substrate. The definition of the type of fermentation results in the need for adequate fermentation conditions such as temperature, pH, medium culture among other parameters which induce the formation of molecules with phytotoxic effects (Ocán-Torres et al. 2024; Camargo et al. 2023). The pathogenicity of some strains is increased by the composition of the medium and the conditions cited (Salvatore et al. 2024), as shown in Table 1.

The composition of the fermentation medium also influences the obtaining of biomolecules. Catabolite repression (CR) is a mechanism used by microorganisms to modulate the production of secondary metabolites and enzymes, through the use of simple sources of carbon and/or nitrogen before investing energy in the hydrolysis of disaccharides or other nutrients. In microorganisms such as the genus *Bacillus* spp., Carbon Catabolite Repression (CCR) preferentially uses glucose as a carbon source. Through the phosphoenolpyruvate: phosphotransferase system (PTS), responsible for transporting and phosphorylating sugar substrates into the cell. The activity of the PTS directly regulates the phosphorylation state of the HPr protein (Histidine protein carrier), a protein that transports phosphates. When there is an abundance of the preferred carbon source, such as glucose, the PTS is actively transporting and phosphorylating the glucose, resulting in a large portion of the HPr in its non-phosphorylated (non-P-HPr) state. It is this non-phosphorylated form of HPr (non-P HPr) that signals the abundance of carbon. Non-P HPr, in conjunction with an intermediate metabolite of glycolysis (usually fructose-1,6-bisphosphate), interacts with the protein CcpA (catabolite control protein A). The resulting complex (non-P CcpA/HPr) binds to specific regulatory sites in DNA, known as Catabolite Responsive Elements (CRE). This link induces the repression of genes that are responsible, for example, for the breakdown and use of more complex carbon sources (Nair and Sarma 2021; Sanchez and Demain 2002).

When using sources such as sucrose (disaccharide), the cell must first produce the enzyme invertase to hydrolyze it into glucose and fructose. This extra step ensures that glucose is released into the medium at a slow, controlled rate (Nair and Sarma 2021). This slow rate of release attenuates CCR, keeping the CcpA signal weak and allowing the biosynthesis pathways of secondary metabolites to operate in parallel with growth, resulting in a higher cumulative production of the biomolecules (Ruiz et al. 2010).

Other bacteria such as *Streptomyces* spp. and *Pseudomonas aeruginosa* have different preferential sources of carbon such as glutamate and succinate, respectively. Different nutrient sources lead to other repressor genes for assimilation of the preferred source of carbon (Ruiz et al. 2010). In the study by Lurthy et al. (2025) the authors found that carbon sources containing fumarate had higher production of metabolites from the fermentation of *Pseudomonas* spp. In addition, when the composition of the medium used fructose and pyruvate, there was an overexpression of a metabolite identified as 2,4-Diacetylphloroglucinol, which can act to inhibit the root development of some weeds such as broomrape.

In fungi, CCR utilizes CreA as a transcription factor that represses the genes of enzymes that utilize carbon disaccharide sources. To activate and deactivate this transcription factor, ubiquitination and phosphorylation steps are involved. Glucose depletion causes the inactivation of the cascade of kinases that detects sugar. CreA is dephosphorylated by phosphatases, which renders it inactive or induces its export from the nucleus to the cytoplasm. CreA in the cytoplasm becomes a target for the ubiquitination apparatus, degrading and inactivating CCR (Adnan et al. 2017; Nair and Sarma 2021).

In Nitrogen Catabolite Repression (NCR), microorganisms exhibit distinct preferences for specific nitrogen sources depending on the species (Adnan et al. 2017). Ammonium ions, glutamate, glutamine, and N-acetylglucosamine are some of the major sources of nitrogen that repress genes for the consumption of secondary nitrogen sources (Nair and Sarma 2021). In the study by Sanchez and Demain (2002) the authors bring a detailed discussion of how different nitrogen sources affect the metabolic pathways for the production of biomolecules.

Trace elements are those substances added to assist in the synthesis of amino acids, energy metabolism and the formation of important cellular structures. Of the main trace elements, magnesium (Mg^2+^) can be highlighted, which acts as a cofactor for the synthesis of several enzymes for microbial growth. Its presence acts in the repair of DNA and RNA, in addition to stabilizing the cell membrane, influencing the permeability and transport of nutrients through it. Magnesium also forms a complex with adenosine triphosphate (ATP), stabilizing it and making it assimilable by enzymes involved in the Krebs cycle (Sanchez and Demain 2002; Ruiz et al. 2010).

Parameters such as pH and temperature also influence the production of enzymes and other metabolites (Kabir and Ju 2023). Changes caused by modifications in the pH of the culture medium affect the catalytic activity of enzymes due to the interaction that occurs between the ionic groups present in the active sites of certain enzymes (Yuwono and Kokugan 2008). These ionic groups have a specific range (acidic or alkaline) to function, so unwanted variations in pH can inhibit enzyme activity, since the active site does not remain in the appropriate ionic form to bind to the substrate (López-González et al. 2024).

The temperature used in fermentation could be near to the environmental temperature where the microorganism was isolated from (Ocán-Torres et al. 2024).

The production of amylase and cellulase through the SbF of *Aspergillus niger* was evaluated as a function of the pH of the fermentation medium (Bellaouchi et al. 2021). The authors observed that the optimal pH condition for amylase production was pH 5.0 (9.53 U/mL) and pH 6.0 for cellulase (4.21 U/mL). For both enzymes, the extreme pH points that the authors evaluated (pH 3.0 and pH 8.0) caused the lowest enzymatic activities, which demonstrates that each enzyme has an optimal pH range, resulting in greater enzymatic activity. This can be justified by the modification of the ionic character of the existing groups on the surface of the enzymes, which consequently also affects the enzyme-substrate bond, hindering saccharification processes of the culture medium (Juárez-Enríquez et al. 2022). Temperature significantly affects the production of enzymes in the growing medium. It is interesting to note that in the data presented in Table 1, there is little use of temperatures above 30 °C. This may be associated with the temperature close to the native habitat of the enzyme, considering that it was obtained from the isolation of microorganisms (Kabir and Ju 2023). Therefore, a more faithful representation of the original biological state can influence higher rates of enzymatic activity in the solution.

Although the studies observed for the construction of this article use this temperature range, the denaturation of enzymes can occur in larger ranges, between 40 and 60 °C, depending on the enzyme (Wiltschi et al. 2020). The denaturation of these molecules can be explained by a physical phenomenon: as there is an increase in temperature, the number of collisions of molecules also increases, altering the thermodynamics of the solution. Thus, chemical bonds such as hydrogen bonds, van der Waals forces, and ionic interactions can undergo changes, modifying the protein structures of the enzyme (Silva et al. 2024). This can cause a change in the active sites and the enzymes are unable to bind to the substrates, causing their denaturation. While high temperatures can cause protein denaturation, low temperatures inhibit the catalysis of reactions because there is not enough energy to increase enzyme activity (López-González et al. 2024). The search for sustainable and low-cost production processes has driven the valorization of agro-industrial residues as substrates for fermentations (De Oliveira et al. 2019; Krein et al. 2023). By using these materials as cultivation media, the concept of circular economy is introduced to the production process, reducing waste generation and contributing to processes with greater economic viability. This practice is directly aligned with the Sustainable Development Goals proposed by the UN (2015), promoting responsible production and consumption, sustainable agriculture, and industrial innovation. Bordin et al. (2018) evaluated the cost reduction for bioherbicide production through the reuse of microalgae biomass for fermentation of *Trichoderma koningiopsis*. The authors observed that the use of microalgae had a significant effect (*p* < 0.05)on the production of enzymes such as cellulase and peroxidase and provided a reduction in production cost related to the culture medium.

## Mechanisms of action of microbial bioherbicides

The application of crude fermented broths can confuse the mechanisms of action caused by herbicides based on microorganisms, since the extract can contain different biomolecules that will act on more than one site of action (Duke 2024). Thus, it is important to evaluate the composition of these broths to understand the influence of the solubilized molecules and their interaction with the weed (Zhang et al. 2025). Some enzymes and secondary metabolites could degrade structural components such as the cell wall and to act on biomolecules essential for the metabolism of target organisms, inhibiting their growth and reproduction (Islam et al. 2024). Understanding these mechanisms is critical to developing more efficient biocontrol strategies.

The classification of the mechanisms of action of synthetic herbicides is regulated by the Herbicide Resistance Action Committee (HRAC), grouping different products that act under the same metabolic process of the plant (Duke 2024). Among the activities caused by herbicides are the inhibition of key enzymes of metabolic pathways, interruption of processes such as photosynthesis and cell division, synthesis of carotenoids, among others (HRAC, 2026; Dayan and Duke 2014). Most bioherbicides do not have a traditional HRAC code (A to Z/1 to 30), as they are usually related to contact damage such as cell wall or membrane destruction (Dayan and Duke 2014; Piasecki 2024). Although it is not considered a bioherbicide, the glufosinate ammonium molecule (Glutamine Synthetase inhibitor), for example, is a compound derived from the degradation of bialaphos, a tripeptide produced by bacteria of the genus *Streptomyces* spp. (Piasecki 2024).

The main mechanism of action observed in studies related to bioherbicides refers to the inhibition of photosystem II (PSII). In this case, the reduction in photosynthetic activity occurs due to the reduction in electron transfer. In the photosystem, electrons from the oxidation of water during the photosynthesis process are excited by the energy captured by pigments (such as chlorophyll and carotenoids). The excited electron is carried by proteins, such as plastoquinones A (Q_A_) and B (Q_B_), which act as electron acceptors. The Q_A_ molecule transfers electrons to Q_B_, which binds to D1 proteins, responsible for converting light energy into chemistry. When a second electron is transferred to Q_B_, the reduced quinone becomes protonated (Q_BH2_), reducing its affinity for the D1 protein. Thus, the active site of D1 is free to bind with other substances. If the bond between D1 and Q_B_ does not occur, there is no electron transfer, blocking photosynthesis and inhibiting other biochemical processes such as CO_2_ fixation and ATP production (Cheaib and Killiny 2025; Chen and Qiang 2017). In addition, an accumulation of energy in chlorophyll molecules due to disruption of electron flow is transferred to molecular oxygen molecules, which initiate the production of reactive oxygen species (ROS). From this point, new biochemical processes can be triggered generating thylakoid lipid peroxidation and leading plant cells to oxidative stress and consequent cell death. Toxins such as Cytochalasin, Tenuazonic acid (TeA) and Gliotoxin are some substances produced by fungal fermentation capable to interrupt electron flow (Chen and Qiang 2017; Guo et al. 2020; Jiang et al. 2022). The TeA, for example, is often found as a secondary metabolite of the fermentation of *Alternaria* spp., a phytopathogenic species common in crops such as vegetables, tomatoes, and potatoes (Chen and Qiang 2017; Cheng et al. 2021; Hoagland and Boyette 2021; Wang et al. 2025).

Carotenoids are another key target in bioherbicide research; they are responsible for safeguarding chlorophyll molecules against oxidative damage induced by ROS (Piasecki 2024). The inhibition of carotenoid biosynthesis, in turn, can be caused by the action of phytotoxic compounds such as Macrocidins (Hubbard et al. 2016), produced by *Phoma macrostoma.* Carotenoids act in the protection of chlorophyll molecules by the chelating action of ROS. When the light intensity is high, chlorophyll molecules are exposed to a greater amount of photons than they are capable of absorbing, resulting in an increase in excitation from singlet chlorophyll to triplet chlorophyll, a phytotoxic compound. Thus, carotenoids end up acting as a sunscreen to prevent the state of excitement of chlorophyll. The presence of macrocidin can cause a reduction in the concentration of carotenoids in the leaves, due to the binding with metals, reducing the activity of enzymes such as phytoene desaturase (PDS), which are key enzymes in the process of carotenoid formation (Hasan et al. 2022; Zhang et al. 2025).

Enzymes obtained by fermentation processes can also act in cellular processes, aiding in the physiological development of the plant. Some enzymes such as superoxide dismutase (SOD), catalase (CAT) and peroxidase (POD) participate in the plant’s defense system whose main function is to eliminate reactive oxygen species (ROS), as a way to protect against an increase in oxidative stress (Radhakrishnan and Lee 2013; Hasan et al. 2022). However, when the oxidation of important components of the plant cell occurs, some enzymes exhibit irregular activity and instead of triggering functions to protect the plant system, they end up suppressing the activity of others, reflecting in the accumulation of hydrogen peroxide inside the cell (Hasan et al. 2022). Thus, an imbalance between stressor compounds and antioxidants is caused, influencing the formation of reactive compounds that are cytotoxic to certain cellular constituents, causing protein denaturation and lipid peroxidation. Other enzymes when produced by pathogenic microorganisms, start to degrade cellular components of the host plant (Ocán-Torres et al. 2024), leading to symptoms such as chlorosis and necrosis.

The main barriers encountered by bioherbicides are the cuticle, cell wall, and plasma membrane in weeds (Piasecki 2024). Its composition varies according to plant species, age and the number of cotyledons (mono or dicots that represent narrow and wide leaves, respectively). Despite these significant differences, the compounds found are mostly cellulose microfibrils, pectin, lignin, proteins, among others (Kubicek et al. 2014).

The leaf cuticle, for example, is a layer composed of a polyester lipid matrix (cutin) and is considered the main barrier between the internal structure and the environment, acting as a protective barrier against biotic and abiotic stresses, and regulating water loss from the leaves (Heredia et al. 2024). Cutin is composed of hydroxy and epoxy fatty acids, which vary from 16 to 18 carbons in its composition. According to Kim and Lee (2006), some microorganisms such as *Fusarium* spp., *Pseudomonas* spp., and *Corynebacterium* spp. can use cutin as a carbon source and excrete cutinolytic enzymes, which will act on the hydrolysis of the ester bonds between cutin chains, leading to a consequent degradation (Arya and Cohen 2022). Therefore, damage to the cuticle structure from the enzyme cutinase could create channels for the absorption of phytotoxic compounds, affecting weed metabolism. In the work by De Oliveira et al. (2019) the authors evaluated the herbicidal potential of a fermented broth of four different species of *Fusarium* spp. for the control of *Conyza s*pp. and *Ipomoea* spp. It was observed that the *Fusarium verticillioides* strain had a greater phytotoxic effect for both plant species, including higher cutinase production (16.44 U/g).

Other enzymes as lipases can also be applied to degrade cuticle (Camargo et al. 2024; Heredia et al. 2024). The cell wall of plants is protected by a fatty acid barrier, acting as a hydrophobic barrier. Lipase, the enzyme responsible for lipid metabolism, can degrade the plasmatic membrane that regulates the entry and exit of substances from the cell. These enzymes hydrolyze lipids into fatty acids and glycerol, causing the disruption of the lipid barrier. The accumulation of glycerol in the cytoplasm can interfere with cellular water balance, increasing osmotic pressure and generating an unfavorable gradient through increased proton flow, which would facilitate the entry of other phytotoxic substances. Regarding fatty acids, they can react with ROS and cause peroxidation of the lipid layer, in addition to interfering with mitochondrial activity and depigmentation of chloroplasts.

Enzymes such as proteases, hydrolyze peptide bonds of protein chains. These enzymes can also be classified into endopeptidases and exopeptidases, each of which acts at a specific place in the peptide chain. For the biocontrol of *Senna obtusifolia*, Hoagland and Boyette (2021) observed that the increase in protease concentration in the fermented broth of *Alternaria cassia* was able to increase infectivity in the weed, contributing to the increase in the degradation of protein structures present in the cell wall.

**Fig. 4:**
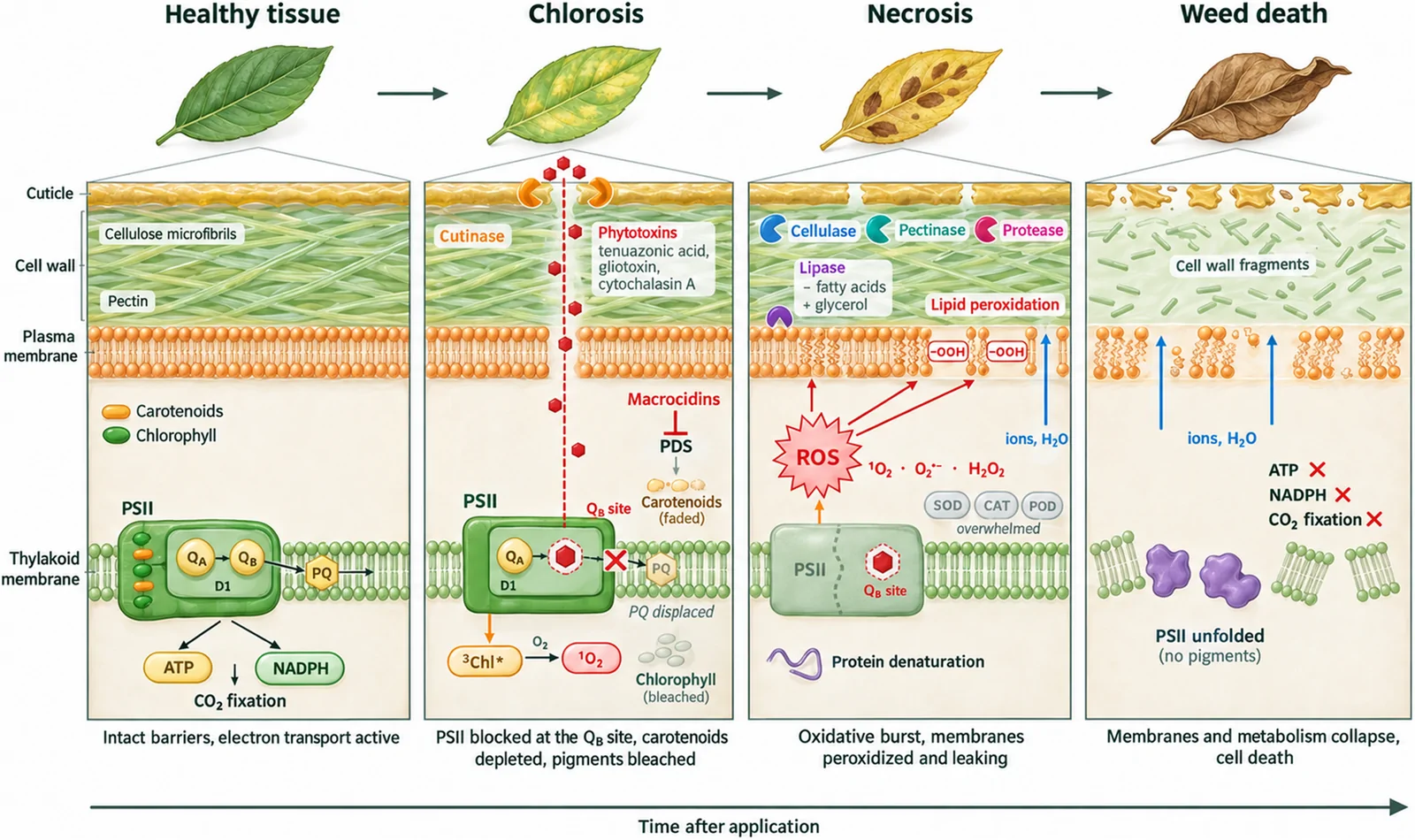
Progression of weed tissue damage induced by microbially derived bioherbicides

Other enzymes such as pectinases, amylases and cellulases are also reported in the literature as important molecules to assist in the degradation of polysaccharides contained in the structure of plants, contributing to the control of plants such as *Amaranthus hybridus*,* Brachiaria plantaginea*,* C. sativus*,* Euphorbia heterophylla*,* Lasiodiplodia pseudotheobromae* and *Senna obtusifolia* (Adetunji et al. 2017; Bordin et al. 2018; Cavalcante et al. 2021; De Oliveira et al. 2019; Hoagland and Boyette 2021; Radhakrishnan and Lee 2013; Stefanski et al. 2020; Ulrich et al. 2021). The biomolecule classes described above, their targets in the plant and the quantitative effects reported in the selected studies are summarized in Table 2, and the progression of these effects over time is illustrated in Fig. 4.

**Table 2:** Classes of microbially derived biomolecules, their targets in the plant and the quantitative phytotoxic effects reported in the selected studies

Biomolecule class	Target	Quantitative effect	Reference
Cell wall-degrading enzymes (cellulase, amylase, peroxidase)	Cell wall polysaccharides	5 mL of extract per plant caused leaf damage in *C. sativus* of 66.67% (7 d) and 75.02% (15 d) for *T. koningiopsis*, and 77.08% and 79.16% for *R. stolonifer*; peroxidase activity of 101.88 and 95.32 U/mL	Cavalcante et al. (2021)
Pectinase	Cell wall pectic polymers	Inoculation with 10⁵ spores/mL increased pectinase activity 1.5-fold in light-grown and 2-fold in dark-grown sicklepod seedlings (72–96 h)	Hoagland and Boyette (2021)
Cutinase	Leaf cuticle (cutin)	Maximum yield of 16.22 U/g of substrate; the formulation with 250 U of cutinase plus bioherbicide gave the highest control of *Conyza* sp. and *Ipomoea* sp.	De Oliveira et al. (2019)
Exopolysaccharides	Leaf surface and foliar tissue	Crude broth (6.62 g/L) caused 100% yellowing; MF retentate (9.18 g/L) caused 30.4% chlorosis, 48.2% necrosis and 21.4% yellowing; lyophilized broth (10.64 g/L) caused 100% total depigmentation in *C. sativus*	Luft et al. (2021); Todero et al. (2020)
Photosynthesis-inhibiting metabolites (cycloheximide, julichromes)	Photosystem II, membrane integrity	GR₅₀ of 1.37 g/L in *D. ciliaris*, with 36% chlorophyll inhibition at 2 g/L; 0.2 mg/mL inhibited shoot and root growth of *A. retroflexus* by 92.31% and 93.71% in pre-emergence	Umurzokov et al. (2023); Ling et al. (2023)

Enzymes or other molecules act on metabolic processes through diffusive and competitive mechanisms. From this, the understanding of the mechanisms of action of bioherbicide molecules becomes even more important, in order to understand how they can be increased by directing enzymes/molecules to specific substrates contained in the structural composition of plants (Wiltschi et al. 2020).

## Protein engineering for bioherbicide optimization

After identifying the mechanisms of action and the genes involved, the application of molecular techniques to optimize the bioherbicide action is important for the development of these bioproducts. These strategies should serve not only to optimize the production of biomolecules on a large scale, but also to improve stability and specificity in the field (Mukhtar et al. 2023).

In the work by Wiltschi et al. (2020) the authors describe that the use of omics tools for the development and obtaining of enzyme-based bioproducts accelerates the understanding of enzymatic mechanisms, making biocatalysis a more competitive alternative compared to conventional chemical processes. Technologies such as metagenomics facilitate the identification of microbial species that may produce a higher concentration of a certain metabolite, including extracellular enzymes. During the sequencing of the genome of a microbial community, RNA transcription is able to identify new functional activities of species that have not yet been explored. Currently, several databases are already available that detail the genome of microbial communities, which facilitates a more in-depth study of microbiology, reducing the time and cost of laboratory procedures that involve extensive analyses.

To understand the dynamics between a biomolecule with bioherbicidal potential and the target plant, the molecular Docking technique can be employed Meng et al. (2011). This computational modeling tool (in silico) simulates the interaction of a small molecule (the ligand, which is the secondary metabolite or enzyme) in a cellular environment, in front of the receptor. A key enzyme or an essential protein of weed metabolism that is to be inhibited (the receptor) is chosen and the interactions of each ligand against the active site of the target enzyme are simulated. The core of docking is the exploration of three-dimensional space to identify all possible ligand orientations and conformations within the protein binding site. For each conformation generated, the software applies a scoring function to estimate the interaction energy between the ligand and the receptor, considering forces such as van der Waals interactions, hydrogen bonds, and electrostatics. The more negative the scoring energy, the better the affinity and the more stable the complex, indicating a potential bioherbicide candidate.

In the work by López-González et al. (2024) the authors used Docking to understand the mechanism of action of pelargonic acid (PA) as a bioherbicide. The modeling suggested that PA interacts with the auxin exporting protein PIN1At. PA is able to bind at the PIN1At site of action in a manner similar to auxin transport inhibitors. Interference with the flow of auxins can stimulate the extrusion of protons by cells, acidifying the cell wall and breaking hydrogen bonds that stabilize the cell wall. As a result, there is an increase in cell expansion and disordered growth of tissues. In the work by Guo et al. (2020) the authors investigated how the fungal compound gliotoxin (GT) can interfere with photosystems II and I. The docking technique demonstrated that GT binds to the Q_B_ site of the PSII D1 protein through hydrogen bonds, replacing plastoquinone, and interrupting the flow of electrons. Docking not only predicts whether a molecule will bind, but how it will bind. By understanding the binding orientation, one can propose structural modifications to the biomolecule to further increase the binding affinity and, consequently, the potential of the bioherbicide.

The gene expression technique is fundamental for bioherbicide biotechnology, as a way to develop new molecules from already established fermentative cultures (Mukhtar et al. 2023). Within this context, techniques such as recombinant proteins and CRISPR are used to manipulate genes with the aim of making microbial cells a kind of “factory” for the production of phytotoxic molecules (Wadhwa et al. 2024).

To maximize the production of certain molecules, the recombinant protein technique is applied to genetically manipulate a cell and induce it to express a gene of interest. The gene responsible for encoding the enzyme or metabolic pathway that produces the phytotoxic biomolecule is identified and isolated from the original microorganism through techniques such as Polymerase Chain Reaction (PCR) (Hamedi and Papiran 2015). This gene is inserted into an expression vector (usually a plasmid) through restriction enzymes and transferred to a host organism that grows rapidly and in large volume, such as the bacterium *Escherichia coli* or yeasts (Mukhtar et al. 2023). The genetically modified host expresses the gene at very high levels, producing the bioherbicide enzyme or the secondary metabolite in pure, economically viable amounts. This final product, called recombinant protein, can then be isolated, formulated, and used as a bioherbicide (Duke et al. 2024).

An *nlp* gene encoding a necrosis and ethylene-inducing peptide 1 (Nep1)-like protein from *Streptomyces cyaneofuscatus* was overexpressed in *E. coli* by Hamedi and Papiran (2015). The *nlp* fragment was amplified via PCR and cloned into a TA vector. To verify heterologous expression, the resulting *E. coli* culture filtrate was topically applied to *Nicotiana tabacum* leaves. Necrotic lesions were observed 48 h post-inoculation, confirming the protein’s biological activity.

Unlike gene expression tools, CRISPR is a technique for gene editing, allowing genetic alterations in segments of DNA responsible for the synthesis of proteins and especially secondary metabolites (Zhang et al. 2025). In addition, the CRISPR/Cas9 technique can be used to activate metabolic pathways or reduce the concentration of unwanted compounds (Wadhwa et al. 2024).

Using the CRISPR/Cas9 technique, Zhao et al. (2022) achieved a maximum production of 504.6 µg/mL of Thaxtomin A, a molecule with bioherbicidal potential produced by *Streptomyces coelicolor* M1154. The native genes of the thaxtomin A cluster were replaced by builder promoters such as the txtED operon, txtABH operon and the txtC gene, causing an improvement in the yield of Thaxtomin A of up to 20 times. By using constitutive promoters, it is ensured that biosynthetic genes are expressed at high levels, regardless of environmental conditions or the state of host cellular regulation.

The integration of these techniques allows exploring the potential of biomolecules and leading to an increase in their production for commercial purposes.

## Challenges and future perspectives

**Fig. 5:**
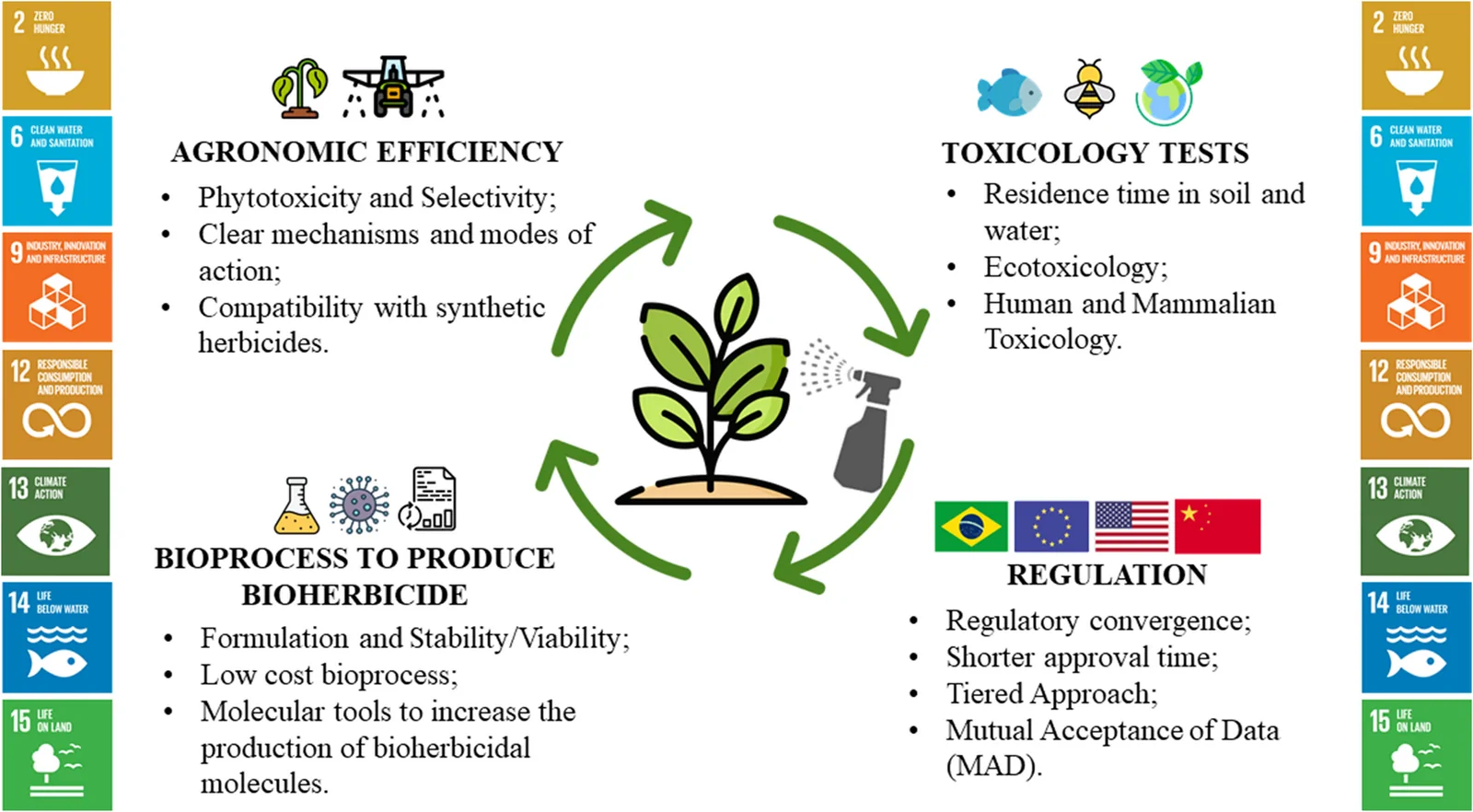
Challenges and perspectives in relation to the production of bioherbicides

Driven by the demand for sustainable agricultural systems, bioherbicides have emerged as a pivotal alternative for weed management. Composed of microorganisms, natural metabolites, or enzymes, these products offer high selectivity and low environmental toxicity, directly aligning with the United Nations Sustainable Development Goals (SDGs). By reducing reliance on conventional agrochemicals, bioherbicides foster the integration of sustainability and productivity, mitigating climate change impacts while promoting soil and ecosystem health. However, despite the favorable outlook, its large-scale application still faces relevant technical and regulatory challenges (Stefanski et al. 2020). Figure 5 demonstrates the main challenges and perspectives related to the production of bioherbicides obtained through the fermentation of microorganisms.

One of the main challenges is related to the formulation and stability of bioherbicides. Unlike conventional chemical herbicides, many bioherbicides are composed of biologically active molecules sensitive to environmental factors such as temperature, UV radiation, pH, and humidity. In addition, products based on microorganisms or enzymes require formulation strategies that ensure viability and biological activity during storage, transportation, and field application (Krein et al. 2023). The proper choice of adjuvants, encapsulants, and controlled-release systems is crucial to increase product shelf life and ensure consistent performance in the agricultural environment (Brun et al. 2022b; Krein et al. 2024). Another challenge is to make these formulations compatible with existing agricultural practices, such as tank mixtures and joint use with synthetic herbicides, without loss of efficiency.

From a production point of view, the scalability of the bioprocess also directly influences the formulation. Low-cost processes, with high productivity and reproducibility, are essential to make the final product economically viable (Mupondwa et al. 2015). In this context, the use of molecular and metabolic engineering tools emerges as an important perspective to increase the production of bioherbicide molecules and reduce variability between batches, facilitating the standardization required for commercial formulations (Wadhwa et al. 2024).

Research into the mechanism of action of bioherbicides is also important, in order to classify them in a similar way to what HRAC brings. In this way, a consortium between herbicides + bioherbicides can be established to optimize the control effect (Bordin et al. 2021). Bioherbicides based on lipid enzymes, for example, could be applied in conjunction with ACCase inhibitors. ACCase’s product, Malonyl-CoA, is a potent inhibitor of CPT-I (carnitine palmitoyltransferase I), a key enzyme in fatty acid oxidation. When ACCase is inhibited through the action of herbicides, Malonyl-CoA levels drop. The drop in Malonyl-CoA alleviates the inhibition of CPT-I, which theoretically increases the oxidation of existing lipids.

Regulatory issues represent another major obstacle to the consolidation of bioherbicides in the market. There are still uncertainties, in many countries, about the clear definition of which tests are necessary for the approval of these products (Duke 2024). Bioherbicides are often evaluated under regulatory frameworks originally developed for chemical pesticides, which can result in requirements that are disproportionate to the actual risk posed by bio-based products (Mordor Intelligence 2025). Among the main points of discussion are human and mammalian toxicology tests, ecotoxicology, as well as impact studies on non-target organisms. The residence time in the soil of bioherbicides based on phytopathogens must be carefully evaluated, in order to prevent it from becoming an inoculum for soil diseases that may later attack crops.

The absence of a standard of criteria makes the registration process difficult and increases costs and approval times, especially for small companies and biotechnology-based startups. On the other hand, regulatory convergence initiatives, such as the adoption of the Tiered Approach and the Mutual Acceptance of Data (MAD), represent a positive outlook. These strategies allow tests to be conducted progressively, based on risk, and data generated in one country can be accepted by other regulatory agencies, reducing redundancies and accelerating market entry (Duke 2024; OECD 2003).

In summary, bioherbicides have great potential to integrate more sustainable agricultural systems, but their broad application depends on advances in more stable and robust formulations, as well as on the construction of clearer, more proportional, and harmonized regulatory frameworks. The alignment between technological development, science-based risk assessment and appropriate regulatory policies will be decisive to transform bioherbicides from a promising solution into a consolidated reality in the field.

## Conclusion

This review examines protein-based microbial bioherbicides by integrating advances in microbial bioprospecting, bioprocess and fermentation strategies, mechanisms of action and protein engineering approaches. The roles of key enzymatic classes involved in weed control, including cutinases, lipases, cellulases, pectinases and proteases, are discussed in relation to their targets within plant structural and metabolic pathways. Particular emphasis is placed on how fermentation conditions and nutrient regulation influence protein production and bioherbicidal efficacy.

Furthermore, emerging molecular strategies such as omics-driven discovery, molecular docking, recombinant protein expression and CRISPR-based metabolic engineering are highlighted as powerful tools to enhance specificity, stability and scalability of protein-based bioherbicides. Finally, the major technological, formulation and regulatory challenges limiting field application and commercialization are critically analyzed, alongside perspectives for regulatory harmonization and integration with existing weed management programs.

Overall, this review positions protein-based microbial bioherbicides as a versatile and expandable platform for next-generation weed control, emphasizing the need for interdisciplinary approaches to bridge fundamental research and agricultural application.

## Data Availability

No datasets were generated or analysed during the current study.
